# BUILDING CAPACITY FOR DEMENTIA CAREGIVER TOOLS AND PRAGMATIC TRIALS THROUGH STAKEHOLDER ENGAGEMENT

**DOI:** 10.1093/geroni/igac059.1283

**Published:** 2022-12-20

**Authors:** Hillary Lum

**Affiliations:** University of Colorado Anschutz Medical Campus, Aurora, Colorado, United States

## Abstract

Caregivers face challenges communicating and coordinating health-related needs on behalf of persons living with dementia (PLWD). This presentation will highlight capacity building activities at UCHealth, a large academic health system in Colorado and Wyoming, to prepare for pragmatic clinical trials to test integrated health informatics tools to improve the care of PLWD and their family caregivers. We will describe foundational activities of: 1) convening diverse patient and family caregivers to identify communication priorities and refine health informatics tools, 2) engaging health system leaders to develop a system-based value proposition, and 3) partnering with health informatics teams to adapt tools (i.e., dementia registry, refined patient portal tools) and implement reports of pragmatic outcomes. Through support as an NIA IMPACT Health Care System Scholar, we will discuss lessons learned related to highlighting the preferences of diverse caregivers, especially related to use of health informatics tools in the context of dementia care.

